# Deep kernel learning improves molecular fingerprint prediction from tandem mass spectra

**DOI:** 10.1093/bioinformatics/btac260

**Published:** 2022-06-27

**Authors:** Kai Dührkop

**Affiliations:** Department of Bioinformatics, Friedrich Schiller University, Jena 07743, Germany

## Abstract

**Motivation:**

Untargeted metabolomics experiments rely on spectral libraries for structure annotation, but these libraries are vastly incomplete; *in silico* methods search in structure databases, allowing us to overcome this limitation. The best-performing *in silico* methods use machine learning to predict a molecular fingerprint from tandem mass spectra, then use the predicted fingerprint to search in a molecular structure database. Predicted molecular fingerprints are also of great interest for compound class annotation, *de novo* structure elucidation, and other tasks. So far, kernel support vector machines are the best tool for fingerprint prediction. However, they cannot be trained on all publicly available reference spectra because their training time scales cubically with the number of training data.

**Results:**

We use the Nyström approximation to transform the kernel into a linear feature map. We evaluate two methods that use this feature map as input: a linear support vector machine and a deep neural network (DNN). For evaluation, we use a cross-validated dataset of 156 017 compounds and three independent datasets with 1734 compounds. We show that the combination of kernel method and DNN outperforms the kernel support vector machine, which is the current gold standard, as well as a DNN on tandem mass spectra on all evaluation datasets.

**Availability and implementation:**

The deep kernel learning method for fingerprint prediction is part of the SIRIUS software, available at https://bio.informatik.uni-jena.de/software/sirius.

## 1 Introduction

Liquid chromatography coupled to mass spectrometry (LC-MS) allows a relatively comprehensive analysis of the metabolome of a biological system. LC-MS analysis can detect hundreds to thousands of metabolites from only small amounts of sample; tandem mass spectrometry (MS/MS) individually fragments the observed metabolites and records their fragment masses. Public repositories containing metabolomic LC-MS/MS data (Haug *et al.*, 2019; Nothias *et al.*, 2020; Sud *et al.*, 2016) are growing quickly, but repurposing these data at a repository scale remains non-trivial.

Structural annotation via MS/MS has historically been carried out by spectral library search; resulting annotations are intrinsically restricted to compounds for which a reference spectrum (usually based on commercially available chemicals) is present in the library. During the last decade, *in silico* methods were developed that allow to search in substantially more comprehensive molecular structure databases (Allen *et al.*, 2015; Brouard *et al.*, 2016; Dührkop *et al.*, 2015; Fan *et al.*, 2020; Schymanski *et al.*, 2017; Verdegem *et al.*, 2016; Wolf *et al.*, 2010). Numerous molecular structure databases exist that may be searched by these *in silico* methods (Kanehisa *et al.*, 2016; Kim *et al.*, 2016; Wishart *et al.*, 2018). Besides searching in databases with ‘established’ molecular structures, *in silico* methods can also be used to search in databases containing hypothetical structures, thereby overcoming the boundaries of known (bio-)chemistry; this recently resulted in the annotation of eleven novel bile acid conjugates (Hoffmann *et al.*, 2022).

The best-performing *in silico* methods (Schymanski *et al.*, 2017) use machine learning to predict a molecular fingerprint of the query compound then use the predicted fingerprint to search in a molecular structure database. Molecular fingerprints are either explicitly predicted using an array of support vector machines (SVMs) (Dührkop *et al.*, 2015; Heinonen *et al.*, 2012; Shen *et al.*, 2014), or implicitly using kernel regression (Brouard *et al.*, 2016, 2017, 2019). Implicit use of molecular fingerprints via Input Output Kernel Regression usually outperforms explicit prediction by a small margin; also, training times become extremely fast. On the downside, running times for searching in large structure databases increase substantially. But most importantly, explicitly predicted fingerprints can be used for related tasks such as compound similarity estimation (Tripathi *et al.*, 2021), compound class prediction (Dührkop *et al.*, 2021) or *de novo* structural elucidation (Stravs *et al.*, 2021), opening up a whole new area of possible research questions.

Whereas the predecessor FingerID exclusively used spectrum-based kernels (Heinonen *et al.*, 2012), all CSI:FingerID variants use multiple kernel learning and combinatorial kernels on fragmentation trees (Böcker and Rasche, 2008; Shen *et al.*, 2014). These combinatorial kernels are responsible not only for the major improvement in search performance (Dührkop *et al.*, 2015) but also for the high generalization performance of the machine learning models (Dührkop, 2018).

Deep neural networks (DNNs) learn an embedding directly from raw data, but this often requires a large amount of training data. Although there are hundred thousands of spectra available in public reference libraries, these are just multiple recordings of a rather small number of compounds. So far, DNNs are mainly used for low-resolution EI-MS data, where transforming the spectrum into a vector is a trivial task (Ji *et al.*, 2020). For high-resolution MS/MS data, DNN methods usually bin the spectrum (Fan *et al.*, 2020).

Kernel methods show great generalization performance even when trained on a small number of spectra, but training them on large datasets is difficult due to cubic time and quadratic space requirement on the number of training data. DNNs, on the other hand, perform well when a large amount of training data is available, and their training time scales linearly with the number of training data when stochastic gradient descent is used. For the metabolite identification task, the training data consist of a large number of spectra measured from a small number of compounds. Training on multiple measurements of the same compound will probably not contribute much to the generalization performance of the predictor but might improve its robustness against noise. Here, we present two kernel-based methods that can utilize the large amount of available spectral training data. We use the Nyström method to embed the kernel into a finite-dimensional feature space. The Nyström method is a common trick to apply SVMs and other kernel methods on large datasets (Cuevas *et al.*, 2020; Lopez-Martin *et al.*, 2019; Meanti *et al.*, 2020; Zhang *et al.*, 2012). The first method is using a linear SVM on this feature embedding. The second method is using a DNN on the Nyström embedding and, thus, combines the strength of kernel learning and deep learning.

Previous research on deep kernel learning focuses mostly on learning better data embeddings with DNNs while relying on the general-purpose radial basis function kernel (Ober *et al.*, 2021; Tossou *et al.*, 2020; Wilson *et al.*, 2016). For the fingerprint prediction task, we already have hand-crafted, highly specialized kernels. Therefore, our deep kernel learning method is using these kernels as input of a DNN.

## 2 Materials and methods

### 2.1 Training data

To train our models, we use a combined dataset from MassBank (Horai *et al.*, 2010), GNPS (Wang *et al.*, 2016) and the NIST 2020 database (National Institute of Standards and Technology). We limit ourselves to MS/MS spectra recorded in positive ion mode, as there are more such spectra available. To the best of our knowledge, this constitutes practically all data available for training machine learning models. Certain libraries contain a large number of simulated fragmentation spectra, usually for certain lipid classes; core fragmentation of these lipids is relatively easy to simulate using a simple rule-based approach. Clearly, simulated spectra do not carry any useful information for training our machine learning models.

Notably, stereoisomers (say, L/D-threose and L/D-erythrose) often result in highly similar fragmentation spectra. The (2D) *structure* of a compound ignores the stereo-configuration for asymmetric centers and double bonds, and only considers atoms and their connectivity. To avoid overestimating a model’s performance, we must ensure *structure-disjoint evaluation*: Data from the same structure must never be present in training and evaluation data. We ensure this by removing all data of the corresponding structures from the training (holdout) or evaluation datasets.

Spectral libraries often contain several spectra of the same structure measured at different collision energies. In the following, we will call the input of the machine learning methods a *compound*, which is either an MS/MS spectrum recorded at a single-collision energy, a merge of several MS/MS spectra from different collision energies, or an MS/MS spectrum recorded at varying collision energies (ramp spectrum).

We use two separate training datasets: The ‘main training’ dataset contains 21 191 structures with 28 000 compounds and 197 832 individual spectra. For this dataset, we merged all spectra of the same structure if they are measured on the same instrument and have the same adduct.

The ‘additional training’ dataset consists of 128 017 compounds. It contains 13 335 additional structures that are not already contained in the ‘main training’ dataset. For the ‘additional training’ dataset, we do not merge any spectra. 47 369 spectra of this dataset are already part of the merged spectra in ‘main training’. The number of recorded spectra per compound can be highly variable: for Isomitraphylline there are 85 spectra in ‘additional training’, for 15-Lipoxygenase Inhibitor I there are only 3 recorded spectra. To avoid that this imbalance affects the training, we weight each structure in the ‘additional training’ dataset by the reciprocal of the square root of its occurrences. By using the square root, we downweight instances with many measurements, but still, recognize that multiple measurements provide additional information.

### 2.2 Molecular fingerprints

Molecular fingerprint prediction is a multi-label classification task on a total of 8925 binary labels, including fingerprints from CDK substructure (Willighagen *et al.*, 2017), PubChem CACTVS (Kim *et al.*, 2016), Klekotha-Roth (Klekota and Roth, 2008), FP3, MACCS, extended connectivity fingerprints (Rogers and Hahn, 2010), and a fingerprint defined from 746 custom SMARTS that describe common patterns and ring structures in biomolecules (Dührkop *et al.*, 2021). Of these 8925 binary labels, we selected 5220 labels that occur in at least 20 training structures for the fingerprint prediction task.

Molecular fingerprints are computed with the Chemical Development Kit version 2.3 (Willighagen *et al.*, 2017). Before computing molecular fingerprints, all molecular structures were standardized using the PubChem standardization procedure (Kim *et al.*, 2016) as described in (Hoffmann *et al.*, 2022). In particular, a canonical tautomeric form was chosen, as solvent, temperature and pH in the sample influence the dominating tautomeric species. Without standardization, a molecular property may be simultaneously present or absent for the same compound.

We do not hash fingerprints (as is it is common for extended connectivity fingerprints or other topological fingerprints); each single label corresponds to a substructure.

### 2.3 Kernels

We use domain-specific combinatorial kernels on fragmentation trees and the probability product kernel on MS/MS spectra as described in Dührkop *et al.* (2019, 2015) and Shen *et al.* (2014). Kernels are combined via multiple kernel learning (Cortes *et al.*, 2012). In total, 14 kernels are selected and combined by the multiple kernel learning.

### 2.4 State-of-the-art

As state-of-art method to evaluate against, we trained an array of SVMs for fingerprint prediction from MS/MS data as described in Dührkop *et al.* (2015). Here, training was carried out solely on the smaller ‘main training’ dataset; computing the kernel for all the training data would require 176 gigabytes of memory just to store the kernel matrix, as well as an immense amount of computing time. We map decision values to posterior probability estimates using Platt probabilities (Platt, 2000), as described in Dührkop *et al.* (2015). The *kernel SVM* has only one hyperparameter per label (the regularization parameter C) which was optimized in a nested cross-validation.

As a second method to evaluate against, we trained a DNN on tandem mass spectra similarly to MetFID (Fan *et al.*, 2020) and conceptually similar to Ji *et al.* (2020): a mass spectrum is transformed into a feature vector by binning all *m*/*z* values. A second feature vector is obtained by subtracting each *m*/*z* value from the precursor mass. Both feature vectors are concatenated and used as input to a DNN. Since our data have high mass accuracy, we used a smaller binning size of 0.005 Da than Fan *et al.* (2020), resulting in 102 093 features. We did the same noise removal procedure as in MetFID but used the square root of relative peak intensities as a feature. Furthermore, we found that we could improve the prediction quality by adding a 50% dropout and larger hidden layers (2500 and 8000 neurons instead of 800 and 600 neurons as in MetFID). To ensure a fair comparison with the kernel-based methods, we also added the molecular formula vector to the DNN input; this information is implicitly encoded in our kernel framework. The molecular formula vector was normalized by dividing each feature by its standard deviation in the training dataset. Peak intensity features are already between 0.0 and 1.0 and were stored in a sparse vector. Hyperparameters of the DNN were optimized on the validation set. In the following, this method is called *spectrum DNN*.

### 2.5 Nyström approximation

The Nyström approximation is a method for approximating an *n *×* n* kernel matrix *K* using only a subset of *m *<* n* columns (Williams and Seeger, 2001). Without loss of generality, we assume that we select the first *m* columns of *K*. We can divide *K* into four blocks

$$K=\left[\begin{array}{cc} K_{AA} & K_{AB}\\ K_{AB}^{T} & K_{BB} \end{array}\right]$$

with *K_AA_* is an *m *×* m* and *K_AB_* an *m *×(*n-m*) submatrix of *K*. The Nyström approximation allows to approximate *K* by using only *K_AA_* and *K_AB_*:

$$K\approx \tilde{K}=\left[\begin{array}{c} K_{AA}\\ K_{AB}^{T} \end{array}\right]K_{AA}^{-1}{\left[\begin{array}{c} K_{AA}\\ K_{AB}^{T} \end{array}\right]}^{T}.$$

As a side effect, this approximation provides a feature embedding for the approximated kernel (Williams and Seeger, 2001). To obtain the feature embedding, we use the eigenvector decomposition of *K_AA_* with $K_{AA}=U\Sigma U^{T}$. Here, *U* is the matrix of eigenvectors of *K_AA_* and Σ is the diagonal matrix of the corresponding eigenvalues. From

$$\begin{array}{c} \tilde{K}=\left[\begin{array}{c} K_{AA}\\ K_{AB}^{T} \end{array}\right]K_{AA}^{-1}{\left[\begin{array}{c} K_{AA}\\ K_{AB}^{T} \end{array}\right]}^{T}=\left[\begin{array}{c} K_{AA}\\ K_{AB}^{T} \end{array}\right]U\Sigma^{-1}U^{T}{\left[\begin{array}{c} K_{AA}\\ K_{AB}^{T} \end{array}\right]}^{T}\\ =\left[\begin{array}{c} K_{AA}\\ K_{AB}^{T} \end{array}\right]U\Sigma^{-\frac{1}{2}}\Sigma^{-\frac{1}{2}}U^{T}{\left[\begin{array}{c} K_{AA}\\ K_{AB}^{T} \end{array}\right]}^{T}\\ =\left(\left[\begin{array}{c} K_{AA}\\ K_{AB}^{T} \end{array}\right]U\Sigma^{-\frac{1}{2}}\right){\left(\left[\begin{array}{c} K_{AA}\\ K_{AB}^{T} \end{array}\right]U\Sigma^{-\frac{1}{2}}\right)}^{T}=\hat{X}{\hat{X}}^{T} \end{array},$$

we can compute the feature embedding $\hat{X}=\left[\begin{array}{c} K_{AA}\\ K_{AB}^{T} \end{array}\right]\Gamma$ with $\Gamma =U\Sigma^{-\frac{1}{2}}$ is the projection matrix that computes the feature map for a given kernel matrix.

During training, the matrix *K_AB_* is the kernel matrix between the ‘main training’ dataset and the ‘additional training’ dataset. For prediction, *K_AB_* is the kernel vector between the ‘main training’ dataset and the test compound.

Using the feature embedding, we can plugin the kernel framework into any machine learning method that accepts a feature vector as input. In the context of kernel learning, the Nyström approximation is usually used to train kernel methods with stochastic gradient descent on large amount of data. Training a kernel SVM scales cubically with the number of training data. When using the Nyström method and stochastic gradient descent, the method scales linearly with the number of training data. However, the eigenvector decomposition has complexity $O(m^{3})$, and the computation of the feature map involves vector-matrix multiplications. The overall complexity of training a Nyström SVM with stochastic gradient descent is $O(m^{3}+m^{2}n)$.

We train the kernel SVM on the complete training dataset using minibatch stochastic gradient descent with the tensorflow library (Abadi *et al.*, 2016). We refer to this machine learning model as *Nyström SVM*. When we multiply Γ with the learned weight matrix (the coefficients of the primal problem) and center the resulting matrix, we get the support vector coefficients for the dual problem. Thus, for prediction, the Nyström SVM does not differ from the kernel SVM and does not require any code changes. We map the Nyström SVM decision values to posterior probability estimates using Platt probabilities (Platt, 2000), as it was done for the kernel SVM.

### 2.6 Combining kernels and DNNs

**Fig. 1.**
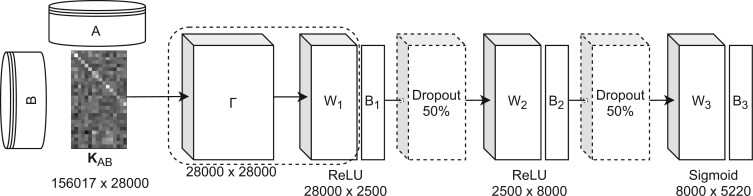
Architecture of deep kernel learning. Here, the input matrix *K_AB_* contains the kernel evaluation on the ‘main training’ dataset (A) against the ‘additional training’ dataset (B). At prediction time, the input matrix would contain the kernel evaluation between the ‘main training’ dataset and the test data. The projection matrix Γ can be multiplied with *W*_1_ into an updated weight matrix; afterwards, the projection matrix is not necessary anymore. ReLU denotes a dense layer with a *rectified linear activation* function

As the second method, we use the feature embedding of the Nyström approximation as input to a DNN with two hidden layers with 2500 and 8000 neurons (Fig. 1). We evaluated the effect of dropout (Srivastava *et al.*, 2014), batch normalization (Ioffe and Szegedy, 2015) and independent-component layer (Chen *et al.*, 2019) and found that using dropout with a rate of 50% works best. We applied *l*_2_ regularization on the output layer with $\lambda =10^{-7}$. We trained the weight parameters with the Adam optimizer (Kingma and Ba, 2015) on a minibatch of size 200 and with a learning rate of $10^{-3}$. We used the sigmoid cross-entropy as a loss function. We trained the model on the complete training data for 25 epochs using the tensorflow library. All hyperparameters of the DNN were optimized on the validation set. We will call this machine learning model *deep kernel learning*, or, in short, deep kernel.

The deep kernel has 131 760 000 parameters. This is less than the 146 160 000 parameters of the kernel SVM and Nyström SVM, but also much less than the 316 992 500 parameters of the DNN on mass spectra.

## 3 Results

### 3.1 Evaluation metrics

We use the Matthews Correlation Coefficient (MCC, also known as Yule’s phi), Bookmakers Informedness (BM, also known as Youden’s J statistic) and Tanimoto (also known as Jaccard Index) as measures of quality for the binary classifiers (Matthews, 1975; Powers, 2003; Tanimoto, 1958). MCC and BM have advantages and disadvantages (Chicco *et al.*, 2021; Zhu, 2020), so we argue it is reasonable to report both. Both measures return values between –1 and + 1, and equal zero for a random classifier. Since we have a multi-label classification problem, we have to average across all MCC and BM values for each single label. Some labels may have a very small number of positive examples or even no positive examples at all. For the latter, neither MCC nor BM is defined. We group all labels with less than 10 positive examples together, sum up the entries in their confusion matrices, and compute a single MCC and BM for them; this strategy is called micro averaging. The Tanimoto is not a measure for the prediction quality of each label, but for the prediction quality of all labels for a single compound.

### 3.2 Hyperparameter estimation

The *C* parameter of the kernel SVM is trained within a nested cross-validation, as it is implemented in CSI:FingerID. The DNNs, however, have much more hyperparameters. For the deep kernel and the spectrum DNN, we evaluated several hyperparameter combinations on the validation set and decided for the hyperparameters that yield the best mean MCC; see Table 1. We found that dropout with a high-dropout rate works better than independent-component layers or batch normalization. Furthermore, increasing the size of the last hidden layer improves the mean MCC, while increasing the size of the first hidden layer is not beneficial. Adding more layers resulted in a degradation of the prediction performance: We trained a deep kernel with 2500, 1500 and 6000 neurons in each hidden layer. Such a network with three hidden layers has a similar number of parameters as the two hidden layer network with 2500 and 8000 neurons in each layer. However, the deep kernel with three hidden layers performed substantial worse with an MCC of 0.5641, compared to an MCC of 0.5909 for the best deep kernel with two hidden layers. The MCC dropped to 0.5164 after adding another intermediate hidden layer with 1500 neurons.

**Table 1.** Mean Matthews Correlation Coefficient (MCC) for different hyperparameter combinations on the validation set

First hidden layer	2500				5000	2500
Second hidden layer	4000				4000	8000
Dropout rate	0%	50%	33%	50%	50%	50%
Batch normalization	Yes	Yes	No			
Mean MCC	0.5642	0.5856	0.5861	0.5899	0.5883	**0.5909**

*Note*: The first two columns describe the number of neurons in the first and second hidden layer. When batch normalization and dropout are applied together, batch normalization happens before the dropout as described in Chen *et al.* (2019). The bold font indicates the highest value in a row.

### 3.3 Cross-validation results

**Fig. 2.**
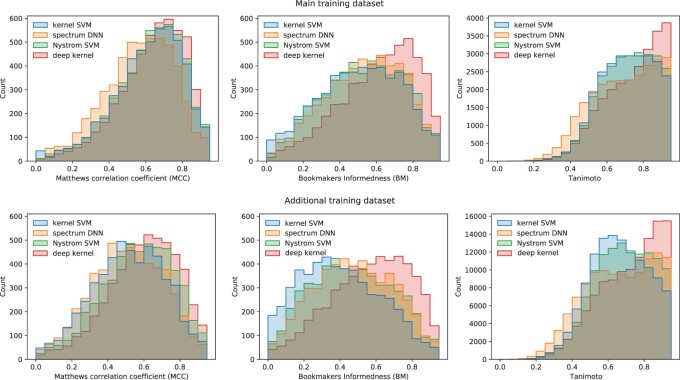
Histogram of MCC (left) and BM (middle) and Tanimoto (right) for individual labels on the ‘main training’ and ‘additional training’ datasets. Predictions are carried out in a structure-disjoint cross-validation. MCC and BM are metrics on the 5220 labels. Tanimoto is a metric on the compounds

We performed a structure-disjoint 5-fold cross-validation for all four methods. The kernel SVM was trained solely on the much smaller ‘main training’ dataset, while the Nyström SVM, spectrum DNN and deep kernel were trained on both training datasets. Yet, the kernel SVM has a higher mean MCC on both training datasets than the spectrum DNN (Table 2). For the mean Tanimoto, it outperforms the spectrum DNN on the smaller training set (0.726 versus 0.708) but not on the larger training set (0.673 versus 0.681). The kernel SVM and the Nyström SVM both have low mean BM on both datasets. The deep kernel learning clearly outperforms all other methods on all three metrics. The deep kernel learning reaches a mean MCC of 0.639 on the ‘main training’ dataset and a mean MCC of 0.598 on the ‘additional training’ dataset. The mean MCC difference between both datasets, one containing high-quality merged spectra and the other containing lower quality single-collision energy spectra, is 0.040 for the deep kernel learning, 0.053 for the Nyström SVM, 0.054 for the spectrum DNN and 0.094 for the kernel SVM. For both datasets, the deep kernel learning predicts more labels with high MCC and BM than the competing methods (Fig. 2). In particular, the deep kernel learning predicts 25.286% of the compounds in the smaller and 22.822% of the compounds in the larger dataset with a Tanimoto above 0.9. To evaluate how much the additional training data contribute to the improved performance of the deep kernel, we retrained the deep kernel on the smaller ‘main training’ dataset. Even with less training data, the deep kernel outperforms all other methods in all benchmarks and is only outperformed by the deep kernel trained on the larger training dataset (Table 2).

**Table 2.** Mean Matthews Correlation Coefficient (MCC), Bookmarker informedness (BM) and Tanimoto on the two cross-validation datasets and on the three independent evaluation datasets

		Kernel SVM small dataset	Spectrum DNN	Nyström SVM	Deep kernel	Deep kernel small dataset
MCC	Main training	0.608	0.563	0.621	**0.639**	0.622
	Additional training	0.513	0.509	0.568	**0.598**	0.580
	Independent merged	0.594	0.573	0.614	**0.656**	0.625
	Agilent single-ce	0.519	0.496	0.546	**0.607**	0.560
	Independent noisy	0.415	0.415	0.451	**0.511**	0.456
BM	Main training	0.505	0.535	0.468	**0.616**	0.534
	Additional training	0.403	0.480	0.410	**0.572**	0.491
	Independent merged	0.512	0.535	0.533	**0.628**	0.593
	Agilent single-ce	0.430	0.453	0.460	**0.574**	0.517
	Independent noisy	0.317	0.371	0.357	**0.472**	0.408
Tanimoto	Main training	0.726	0.708	0.730	0.766	**0.768**
	Additional training	0.673	0.681	0.704	0.744	**0.745**
	Independent merged	0.683	0.668	0.695	**0.731**	0.708
	Agilent single-ce	0.651	0.629	0.664	**0.710**	0.681
	Independent noisy	0.598	0.595	0.620	**0.661**	0.624

*Note*: Nyström SVM and deep kernel are the two methods introduced in this article, whereas kernel SVM and spectrum DNN are the methods we evaluate against. ‘deep kernel small dataset’ refers to the deep kernel method trained solely on the ‘main training’ dataset. The kernel SVM is trained on ‘main training’, too; all other methods are trained on ‘main training’ and ‘additional training’. The bold font indicates the highest value in a row.

### 3.4 Independent evaluation datasets

For further evaluations, we used three independent datasets. The CASMI 2016 evaluation dataset is the positive ion mode data from the CASMI 2016 contest (Schymanski *et al.*, 2017). MS/MS spectra were measured on a Q Exactive Plus Orbitrap (Thermo Fisher Scientific) with 20/35/50 higher-energy C-trap dissociation nominal collision energies. MS/MS data of 127 compounds measured in positive ion mode were provided as part of the contest. Fragmentation spectra from different collision energies were merged. We removed all structures from the CASMI 2016 dataset from both training datasets to ensure that training and evaluation datasets are structure-disjoint.

The Agilent evaluation dataset is the commercial MassHunter Forensics/Toxicology PCDL library (Agilent Technologies, Inc.) with 3243 structures and 3462 independent MS/MS measurements, all measured on an Agilent QTOF instrument with CID fragmentation. Unlike the commercially available library, these mass spectra were not curated. From these spectra, 973 structures were not already part of our training data and were selected for the evaluation.

The WEIZMASS evaluation dataset contains MS/MS data from a structurally diverse set of 3540 plant metabolites, isolated from more than 1400 different plant species (Shahaf *et al.*, 2016). MS/MS data were recorded in ramp mode using collision-induced dissociation fragmentation. We selected 634 compounds from this dataset for evaluation, because the remaining structures were already part of our training dataset.

In total, all independent datasets contain 1734 compounds and 1609 structures. Evaluations of *in silico* methods are often carried out using merged or ramp fragmentation spectra (Schymanski *et al.*, 2017), as these carry the most information. Here, we also evaluate our method’s power if query spectra are recorded at a single-collision energy, since LC-MS/MS datasets are usually recorded in this way. The CASMI 2016 dataset is only available with merged spectra. Similarly, WEIZMASS spectra were recorded as ramp spectra, and no individual collision energy spectra are available. For most compounds in the Agilent dataset, three collision energies (10 eV, 20 eV and 40 eV) were recorded individually. Some compounds were also measured with 1 eV, 4 eV or 8 eV.

The ‘independent merge’ dataset consists of the 1734 compounds from all three independent datasets; spectra of different collision energies are merged together. The ‘Agilent single-ce’ dataset contains the 2977 individual spectra recorded at a single-collision energy from the Agilent dataset. Fragmentation spectra in reference libraries often have much better quality (more signal peaks, fewer noise peaks, better signal-to-noise) than fragmentation spectra from a biological LC-MS/MS run. To simulate this effect in our reference datasets, we ‘added noise’ to each fragmentation spectrum. We use the method of Hoffmann *et al.* (2022) which modifies peak intensities, removes certain peaks and adds ‘noise peaks’. The method avoids simulating noisy spectra that can easily be spotted as artificial: For example, adding noise peaks with (uniform) random mass will result in spectra notably different from experimental ones; so, noise peaks are instead given masses randomly drawn from other measured spectra. The ‘independent noisy’ dataset consists of 4364 compounds from CASMI-2016, WEIZMASS and the single-collision energy spectra from Agilent with noise added according to Hoffmann *et al.* (2022).

Although the three datasets are structural disjoint and independent of the training datasets, they are not mutually independent. Instead, the three datasets represent three different scenarios: having high-quality library spectra, having spectra measured at single-collision energy, and having low quality and noisy spectra.

### 3.5 Fingerprint prediction

**Fig. 3.**
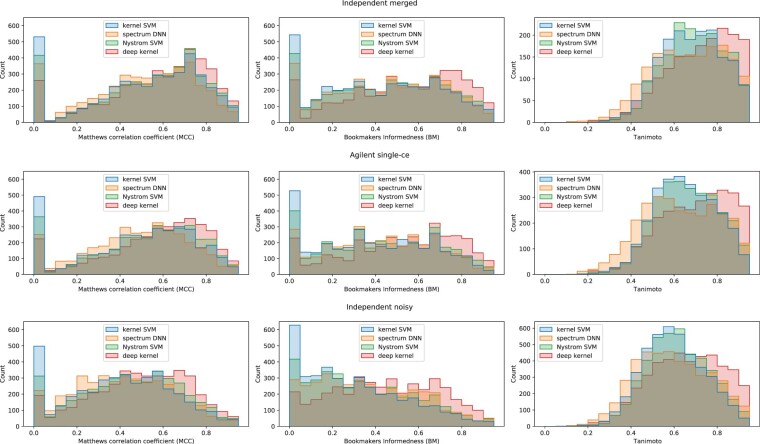
Histogram of MCC (left) and BM (middle) and Tanimoto (right) for individual labels on the ‘independent merge’, ‘Agilent single-ce’, and ‘independent noisy’ datasets. MCC and BM are metrics on the 5220 labels. Tanimoto is a metric on the compounds. Undefined MCC and BM values (for label that do not occur in the dataset) are left out

Again, we evaluate the quality of the molecular fingerprint predictions using MCC, BM and Tanimoto as evaluation metrics (Table 2). We found that on all evaluation datasets, the deep kernel learning clearly outperforms the kernel SVM in all three metrics (Fig. 3). For the ‘independent merge’ dataset, the deep kernel has a mean MCC of 0.656 and a mean BM of 0.628. On the same data, the kernel SVM has a mean MCC of 0.594 and a mean BM of 0.512. The gap between deep kernel and kernel SVM increases with decreasing quality of the data: For the ‘Agilent single-ce’ dataset, the mean MCC and BM is 0.607 and 0.574 for the deep kernel and 0.519, 0.430 for the kernel SVM. The ‘independent noisy’ dataset has the lowest quality spectra; for this dataset, deep kernel learning has a mean MCC of 0.511 and a mean BM of 0.472. The kernel SVM has a mean MCC of 0.415 and a mean BM of 0.317.

### 3.6 Structure database search

For the task of metabolite identification, we search the predicted molecular fingerprints in our in-house mirror of the *PubChem structure database* (Kim *et al.*, 2016). PubChem was downloaded at January 16, 2019 and contains 97 168 905 compounds, and 77 190 484 unique covalently bonded structures with mass up to 2000 Da.

When searching in a structure database, only the exact structure is regarded as correct. Recall that establishing the stereochemistry of a compound from fragmentation spectra is beyond the power of automated search engines and, hence, ignored in evaluations. As scores, we evaluate the covariance score from Ludwig *et al.* (2018), and the Tanimoto score suggested by Laponogov *et al.* (2018) and Ji *et al.* (2020). For a query compound, we assume to know its molecular formula, and we obtained candidates from the structure databases using this molecular formula.

**Fig. 4.**
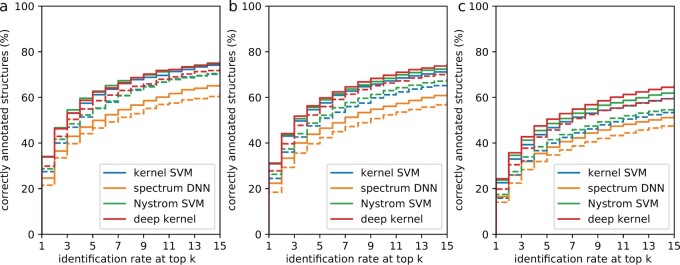
Identification rates on the ‘independent merge’ (**a**), ‘Agilent single-ce’ (**b**) and ‘independent noisy’ (**c**) datasets when using the covariance scoring (solid lines) and the Tanimoto scoring (dashed lines). We report the percentage of instances where the correct structure was identified in the top *k*, for varying *k*

In Figure 4, we report the identification rates on PubChem. The identification rate is the fraction of compounds for which the correct structure is found within the *k* highest-ranked candidates of the database search. A compound is correctly annotated if its structure is the candidate with the highest score. Again, we found that the deep kernel learning and the Nyström SVM perform better than competing methods when the data quality deteriorates. For the ‘independent merge’ dataset, the improvement in correct identifications from using the kernel SVM to using deep kernel learning is 0.119 percentage points. This improvement is higher on the ‘Agilent single-ce’ dataset (0.197 percentage points) and on the ‘independent noisy’ dataset (1.720 percentage points). When using the Tanimoto scoring instead of the covariance scoring, the difference between kernel SVM and deep kernel learning becomes more apparent (Fig. 4). For all scorings and all datasets, the spectrum DNN performs substantially worse than all other methods. For the ‘independent merge’ dataset and the covariance scoring, the spectrum DNN correctly identifies 24.635% of the compounds (33.894% for the kernel SVM, 34.0134% for deep kernel learning and 34.095% for the Nyström SVM).

## 4 Discussion

Both the Nyström approximation and deep kernel learning considerably improved molecular fingerprint prediction. This improvement is higher when the data quality is low, as it is the case for the ‘additional training’ and the ‘independent noisy’ datasets. Both methods become more robust to noise when trained on a large number of spectra, even though these spectra are only duplicate measurements of structures that are already part of the training data. This becomes particularly noticeable when comparing the results of the deep kernel trained on the small dataset with those of the deep kernel trained on the full training dataset. The latter performs substantially better on noisy spectra. The deep kernel learning outperforms the Nyström SVM in most evaluations. A deep architecture together with the dropout regularization technique yields higher MCC, BM and Tanimotos on all evaluation datasets. This is remarkable, considering that both methods work on exactly the same input. It is noteworthy that the kernel SVM consistently performs worse than all other methods in the BM metric. Bookmarker informedness is known to behave equally well even with highly imbalanced data (Zhu, 2020). Thus, it is possible that the kernel SVM is not capable of learning these very rare labels, while the deep kernel can learn more local and non-linear decision boundaries for rare labels.

Unfortunately, these substantial improvements in fingerprint prediction quality do not translate into higher database search identification rates. This is not as surprising as it may seem: Currently, the most severe limitation restricting performance improvements are the available training data. Individual measurements of the same structure—at different collision energies or on different instruments—increase the available information, as we have demonstrated both for Nyström approximation and deep kernel learning. But a 10-fold difference between spectra and structures does not correspond to 10-fold more information: In fact, all spectra recorded at different collision energies from one compound, carry only slightly more information than a single ramp spectrum. Be reminded that only the exact structure was regarded as correct; yet, small structure modifications are hard and potentially impossible to tell apart using MS/MS data alone. This is an intrinsic limitation of small molecule MS/MS; yet, such incorrect annotations may contain viable structure information.

One advantage of deep kernel learning is that it learns all labels together and can thus exploit dependencies between labels. This is noticeable in the high number of compounds predicted by deep kernel learning with almost optimal Tanimoto. The kernel SVM, on the other hand, learns each label independently. It appears that the covariance scoring, which downweights labels that provide little additional information, cancels out this advantage. This might explain why the deep kernel learning improves identification rates for the Tanimoto scoring but shows only modest improvements for the covariance scoring.

The relatively good performance of the spectrum DNN on the three evaluation metrics (MCC, BM and Tanimoto) indicates that the DNN is also capable of learning these label dependencies. However, when searching in structure databases, the spectrum DNN performs significantly worse than all other methods, including the kernel SVM. In fact, the difference between spectrum DNN and kernel SVM seems comparable to the difference between kernels on spectra and kernels on fragmentation trees in Dührkop (2018). However, the deep kernel learning performs well on all three fingerprint prediction metrics as well as in the database search, thus combining the strengths of both approaches.

The improvements in fingerprint prediction performance become important as soon as we leave the application of structure database search: For compound similarity estimation (Tripathi *et al.*, 2021), compound class prediction (Dührkop *et al.*, 2021) and *de novo* structure elucidation (Stravs *et al.*, 2021), we cannot rely on the ‘correctional power’ of a structure database. We expect that our deep kernel learning method will greatly improve these and many other methods that rely on the prediction of molecular fingerprints. Deep kernel learning will replace the kernel SVM in SIRIUS 5.0.

For future development, we see a great potential in pre-training the hidden layers of the deep kernel using variational autoencoders (Kingma and Welling, 2014) or kernel autoencoders (Laforgue *et al.*, 2019), as well as using semi-supervised learning methods, such as self-training (Lee *et al.*, 2017). There are millions of unlabeled spectra in public repositories that can be used to learn a better data embedding. Millions of structures in structure databases can be utilized for learning the relationships and interactions between the labels.
